# Patterns of PET-positive residual tissue at interim restaging and risk of treatment failure in advanced-stage Hodgkin’s lymphoma: an analysis of the randomized phase III HD18 trial by the German Hodgkin Study Group

**DOI:** 10.1007/s00259-023-06431-w

**Published:** 2023-09-22

**Authors:** Justin Ferdinandus, Lutz van Heek, Katrin Roth, Markus Dietlein, Hans-Theodor Eich, Christian Baues, Peter Borchmann, Carsten Kobe

**Affiliations:** 1 Department I of Internal Medicine, Center for Integrated Oncology Aachen Bonn Cologne Duesseldorf, University of Cologne, Medical Faculty and University Hospital Cologne, Gleueler Straße 269-273, 50935 Cologne, Germany; 2 German Hodgkin Study Group (GHSG), Cologne, Germany; 3 Department of Nuclear Medicine, University Hospital Cologne, Cologne, Germany; 4 Department of Radiation Oncology, University Hospital Münster, Münster, Germany; 5 Department of Radiotherapy and Cyberknife Center, University Hospital Cologne, Cologne, Germany; 6 Department of Radiooncology, Marienhospital Herne, Ruhr University Bochum, Bochum, Germany

**Keywords:** PET, Hodgkin’s lymphoma, HD18 trial

## Abstract

**Purpose:**

Response-adapted treatment using early interim functional imaging with PET after two cycles of chemotherapy (PET-2) for advanced-stage Hodgkin’s lymphoma (AS-HL) is the standard of care in several countries. However, the distribution of residual metabolic disease in PET-2 and the prognostic relevance of multiple involved regions have not been reported to date.

**Methods:**

We retrospectively analyzed data from all PET-2-positive patients included in HD18. Residual tissue was visually compared with reference regions according to the Deauville score (DS). PET-2 positivity was defined as residual tissue with uptake above the liver (DS4). PFS was defined as the time from staging until progression, relapse, or death from any cause, or to the day when information was last received on the patient’s disease status and analyzed using Kaplan-Meier and Cox regressions. Comparisons were made between patients with 1–2 and >2 positive regions in PET-2 as well as patients without PET-2-positive regions randomized into comparator arms of HD18.

**Results:**

Between 2008 and 2014, 1964 patients with newly diagnosed AS-HL were recruited in HD18 and randomized following their PET-2 scan. Of these, 480 patients had a positive PET-2 and were eligible for this analysis. Upper and lower mediastinum in almost half of all patients: 230 (47.9%) and 195 (40.6%), respectively. 372 (77.5%) of patients have 1–2 positive regions in PET-2. 5y-PFS for patients with 1–2 regions was 91.7% (CI95: 88.7–94.6) vs. 81.8% (CI95: 74.2–90.1) for those with >2 regions with a corresponding hazard ratio (HR) of 2.2 (CI95: 1.2–4.0). Compared with patients without PET-2-positive disease receiving 6–8 cycles of chemotherapy, patients with 1–2 had a higher risk for a PFS event (HR 1.35; CI95 0.81–2.28), but it was not statistically significant (*p*=0.25). Patients with >2 PET-2-positive lesions had a significantly higher risk (HR 2.95; CI95: 1.62–5.37; *p*<0.001).

**Conclusion:**

PET-2-positive residuals of AS-HL are mostly located in the mediastinum, and a majority of patients have few affected regions. The risk of progression was twofold higher in patients with more than two positive regions in PET-2.

## Short communication

Response-adapted treatment using early interim functional imaging with PET after two cycles of chemotherapy (PET-2) for advanced-stage Hodgkin’s lymphoma (AS-HL) was studied in several academic trials [1–3]. The randomized phase III HD18 trial introduced PET-2-adapted chemotherapy with eBEACOPP and demonstrated that reduction of chemotherapy in PET-2-negative patients is possible without loss of efficacy [1]. PET-2 response is commonly summarized using the Deauville score (DS) [4]. However, the distribution of residual metabolic disease following two cycles of chemotherapy and the prognostic relevance of multiple involved regions in PET-2 have not been reported to date.

Therefore, this study aims to describe the patterns of PET-2-positive residual tissue in HD18 and determine whether multifocal residual disease is associated with inferior progression-free survival (PFS) as compared to uni- or oligofocal disease. We retrospectively analyzed data from all PET-2-positive patients included in HD18. Residual tissue was visually compared with reference regions according to the DS. PET-2 positivity was defined as residual tissue with uptake above the liver (DS4) [5]. PET-2-positive regions were compared to PET after six or eight cycles (EOT-PET) and staging at relapse or progression. Staging after PFS was defined as the time from completion of staging until progression, relapse, or death from any cause, or to the day when information was last received on the patient’s disease status. The prognostic relevance of the remaining regions was first tested using Cox regression of a log-scaled number of PET-2-positive regions as a continuous variable. Log-scaling was done for non-normal distribution. The cohort was then split using >1, >2, and >3 as cutoffs. Comparisons were analyzed using log-rank comparisons and Cox regressions of categorized variables. The study was conducted in accordance with the Declaration of Helsinki and was approved by the review boards of the participating sites. Informed consent was obtained from all individual participants included in the study. The study was registered at www.clinicaltrials.gov as NCT00515554.

**Fig. 1:**
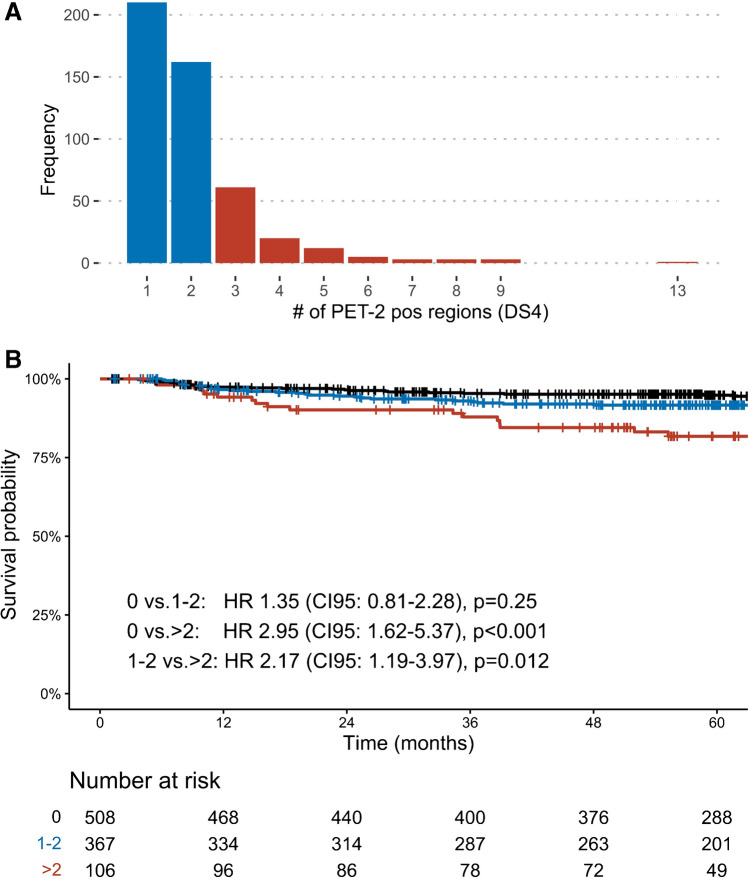
Total number of regions involved (**A**) and Kaplan Meier plots of progression-free survival grouped by number of regions in PET-2 (**B**). DS, Deauville Score; HR, hazard ratio; CI95, 95% confidence interval; patients without PET-2-positive regions are from the respected standard arm of HD18 (pre + post amendment) and are included for reference

Between May 2008 and July 2014, 1964 patients aged 18–60 years with newly diagnosed AS-HL were recruited in HD18 and had an available PET-2 scan. Of these, 480 patients were rated as PET-2-positive (DS4) and were therefore eligible for this analysis. Baseline characteristics are stated in Table 1. The upper and lower mediastinum was involved in almost half of all patients with positive residues: 230 (47.9%) and 195 (40.6%), respectively, see Table 2. A majority of patients had few positive regions in PET-2; 210 (43.8%) had one positive region, and 1–2 or 1–3 involved regions were observed in 372 (77.5%) and 433 (90.2%) patients, respectively. Figure 1A shows a cumulative bar graph of the total number of lesions involved. Among 135 patients with positive EOT-PET, 95 (70.4%) were positive in regions already detected in PET-2, 27 (20.0%) were positive both in and outside PET-2-positive regions, and 13 (9.6%) were positive only outside of previously detected regions.

**Table 1:** Patient characteristics

Characteristic	PET-2 (DS4)-positive *N* (%)	HD18 entire cohort *N* (%)
Trial arm		
A (8× eBEACOPP)	116 (24.2)	217 (11.1)
A6 (6× eBEACOPP)	237 (49.4)	506 (25.7)
B (8× R-eBEACOPP)	120 (25.0)	217 (11.1)
Withdrawn from ITT after PET-2	7 (1.5)	19 (0.9)
Age		
18–19	40 (8.3)	147 (7.5)
20–29	176 (36.7)	722 (36.7)
30–39	131 (27.3)	472 (24.1)
40–49	86 (17.9)	347 (17.6)
50–59	47 (9.8)	257 (13.1)
Sex		
Female	191 (39.8)	761 (38.7)
Male	289 (60.2)	1184 (60.3)
GHSG risk factor		
Large mediastinal mass	193 (40.2)	561 (28.5)
Extranodal involvement	134 (27.9)	379 (19.3)
3 or more Areas involved	389 (81.0	1680 (85.6)
Elevated ESR	326 (67.9)	1239 (63.1)
Ann arbor stage		
II	105 (21.9)	282 (14.4)
III	186 (38.8)	955 (48.6)
IV	189 (39.4	708 (36.0)
IPS		
0–1	33 (6.9)	599 (30.5)
2–3	291 (60.6)	1.035 (52.7)
4–7	156 (32.5)	519 (26.4)

*DS*, Deauville score; *ITT*, intention to treat; *GHSG*, German Hodgkin Study Group; *IPS*, international prognostic score

**Table 2:** PET-2-positive regions

Region	PET-2-positive *N* (%)
Upper mediastinum	230 (47.9)
Lower mediastinum	195 (40.6)
Spleen	45 (9.4)
Lung hilum (right)	40 (8.3)
Bone	34 (7.1)
Axillary (right)	31 (6.5)
Lung hilum (left)	30 (6.3)
Lung (right)	30 (6.3)
Infraclavicular (right)	29 (6)
Supraclavicular (left)	28 (5.8)
Axillary (left)	28 (5.8)
Cervical (left)	25 (5.2)
Cervical (right)	22 (4.6)
Paraaortal	22 (4.6)
Other	21 (4.4)
Infraclavicular (left)	19 (4)
Lung (left)	19 (4)
Iliacal (right)	18 (3.8)
Supraclavicular (right)	15 (3.1)
Iliacal (left)	15 (3.1)
Inguinal/femoral (right)	15 (3.1)
Liver hilum	9 (1.9)
Inguinal/femoral (left)	9 (1.9)
Upper cervical/nuchal/submandibular (right)	8 (1.7)
Mesenterial	8 (1.7)
Liver	7 (1.5)
Bone marrow	7 (1.5)
Upper cervical/nuchal/submandibular (left)	5 (1)
Coelical	3 (0.6)
Splenic hilum	3 (0.6)
Waldeyers ring (right)	1 (0.2)
Waldeyers ring (left)	1 (0.2)
Pleura	0 (0)
Pericardium	0 (0)

At a median follow-up of 61 months, PFS events were recorded in 48 (10%) patients of the entire cohort, translating to a 5y-PFS rate of 88.9 (CI95: 86.0–91.9). More than half of these had involved regions both inside and outside of PET-2 (*n*=26, 54.2%). Detailed comparisons between PET-2 and EOT-PET and staging at recurrence can be found in Tables 3 and 4. Comparison of one versus more than one region was statistically not significant with a HR of 1.73 (CI95: 0.9–3.2). Among the 372 patients with 1–2 PET-2-positive regions, 30 (9.9%) experienced a PFS event. Accordingly, 5y-PFS for patients with 1–2 regions was 91.2% (CI95: 88.2–94.2) vs. 81.2% (CI95: 73.6–89.5) for those with more than 2 regions with a corresponding hazard ratio of 2.1 (CI95: 1.2–3.8). Compared with patients without PET-2-positive disease receiving 6–8 cycles of chemotherapy (5y-PFS 94.8; CI95 92.8–96.8), patients with 1–2 had a higher risk for a PFS event (HR 1.35; CI95 0.81–2.28), which was not statistically significant (*p*=0.25). Patients with >2 PET-2-positive lesions had a significantly higher risk (HR 2.95; CI95: 1.62–5.37; *p*<0.001). Higher numbers of PET-2-positive regions as a numeric variable are associated with a higher risk of relapse/progression according to Cox regression analysis (HR 1.2 increase per region; CI95: 1.1–1.3). Figure 1B illustrates the outcomes of patients with 0, 1–2 vs. more than 2.

**Table 3:** Comparison of PET-2 and EOT-PET

	Location of EOT-PET-positive regions			EOT-PET-negative (*n*=233)	No EOT-PET (*n*=112)
	Inside PET-2 (*n*=95)	Outside PET-2 (*n*=13)	both (*n*=27)		
1-2 regions (*n*=372)	66 (17.7)	11 (3.0)	19 (5.1)	185 (49.7)	91 (24.5)
>2 regions (*n*=108)	29 (26.9)	2 (1.9)	8 (7.4)	48 (44.4)	21 (19.4)

*EOT*, end of treatment (i.e., after 6 or eight cycles of chemotherapy). Inside PET-2 and outside PET-2 imply that patients only had positive regions in EOT-PET that were already positive in PET-2 (inside PET-2) or that were all negative in PET-2 (outside PET-2)

**Table 4:** Comparison of PET-2 and staging at recurrence

	Localization at recurrence				No recurrence (*n*=432)
	Inside PET-2 (*n*=5)	Outside PET-2 (*n*=12)	Both (*n*=26)	Missing data (*n*=5)	
1–2 regions (*n*=372)	2 (0.5)	9 (2.4)	15 (4.0)	4 (1.1)	342 (91.9)
>2 regions (*n*=108)	3 (2.8)	3 (2.8)	11 (10.2)	1 (0.9)	90 (83.3)

Inside PET-2 and outside PET-2 imply that patients only had involved sites of disease at recurrence that were already positive in PET-2 (inside PET-2) or that were all negative in PET-2 (outside PET-2)

Summarizing interim response by reporting the single “hottest” lesion may omit relevant prognostic information. In our study, we find that patients with 1–2 DS4 regions have a comparable outcome to PET-negative patients [1]. Instead, patients with more widespread residual disease have significantly inferior PFS. Our previous work has shown that the likelihood of PET-2-positive disease is significantly higher in patients with large mediastinal mass [5] and high tumor burden [6]. Here, we observe that most patients in fact have residual PET-2-positive disease in the mediastinum. This points to a critical question: does a positive interim scan reflect biologically less chemosensitive or even refractory lymphoma? or does it point towards locally reduced efficacy, e.g., in bulky disease? While the former provides a rationale for treatment escalation in the form of more intense or prolonged systemic treatment, the latter could advocate for focal approaches such as irradiation.

Potentially, our results indicate an adverse prognosis of the higher volume of residual disease. However, the analyses in this manuscript are based on visual assessments (DS) and thus may be subject to inter-reader variability. Baseline PET-CT was only available in a minority of patients in HD18 as it was not a mandatory procedure and not reimbursed in Germany during the recruitment phase of the trial. Future studies are needed to define the role of quantitative imaging biomarkers of baseline PET and PET-2 as prognostic biomarkers in the context of interim restaging of AS-HL. Recent publications in large B-cell lymphoma have already demonstrated metrics such as the metabolic tumor volume or maximum lesion distance to be prognostic for PFS and overall survival in baseline and interim staging [7]. While these may also be relevant in AS-HL, the event rates in this entity are lower, especially with eBEACOPP-based treatment, which necessitates larger cohorts with sufficient power to detect relevant prognostic biomarkers. Besides imaging biomarkers, there is growing interest in the use of longitudinal measurements of circulating tumor DNA (ctDNA) for the assessment of minimal residual disease (MRD) in Hodgkin’s lymphoma during treatment. In principle, MRD could be used to separate patients with AS-HL and PET-2-positive residuals that may still have a good prognosis from those requiring treatment intensification. Two separate studies have reported nearly perfect prediction of treatment failure by MRD and PET-2, concluding that both could complement one another [8, 9].

Our study comes with limitations. First, its retrospective design and the lack of a separate validation cohort call for prospective validation in other AS-HL trials. Second, all patients were treated with two cycles of eBEACOPP before interim PET; therefore, our results may not entirely be transferable to patients starting with ABVD. While this analysis highlights the prognostic relevance of the extent of residual disease in patients with AS-HL, the biological determinants of this rather insufficient response are unknown. Third, there is the absence of histological confirmation of PET-2-positive disease. By definition, it was located in sites with initial disease manifestation and with present residual tissue in CT scans; however, non-lymphoma cannot be entirely excluded.

In summary, PET-2-positive residuals of AS-HL are most often located in the mediastinum. A majority of patients have few affected regions and 5y-PFS comparable to PET-negative patients. However, the risk of relapse or progression was twofold higher in those with more than two positive regions in PET-2.

## Data Availability

The datasets generated during and/or analyzed during the current study are available from the corresponding author on reasonable request.
